# Comparison between proton boron fusion therapy (PBFT) and boron neutron capture therapy (BNCT): a Monte Carlo study

**DOI:** 10.18632/oncotarget.15700

**Published:** 2017-02-25

**Authors:** Joo-Young Jung, Do-Kun Yoon, Brendan Barraclough, Heui Chang Lee, Tae Suk Suh, Bo Lu

**Affiliations:** ^1^ Department of Biomedical Engineering and Research Institute of Biomedical Engineering, College of Medicine, Catholic University of Korea, Seoul, Korea; ^2^ Department of Radiation Oncology, University of Florida, Gainesville, FL, USA; ^3^ Department of Biomedical Engineering, J. Crayton Pruitt Family, University of Florida, Gainesville, FL, USA; ^4^ Weldon School of Biomedical Engineering, Purdue University, West Lafayette, IN, USA

**Keywords:** proton boron fusion therapy, boron neutron capture therapy, Monte Carlo simulation, bragg-peak

## Abstract

The aim of this study is to compare between proton boron fusion therapy (PBFT) and boron neutron capture therapy (BNCT) and to analyze dose escalation using a Monte Carlo simulation. We simulated a proton beam passing through the water with a boron uptake region (BUR) in MCNPX. To estimate the interaction between neutrons/protons and borons by the alpha particle, the simulation yielded with a variation of the center of the BUR location and proton energies. The variation and influence about the alpha particle were observed from the percent depth dose (PDD) and cross-plane dose profile of both the neutron and proton beams. The peak value of the maximum dose level when the boron particle was accurately labeled at the region was 192.4% among the energies. In all, we confirmed that prompt gamma rays of 478 keV and 719 keV were generated by the nuclear reactions in PBFT and BNCT, respectively. We validated the dramatic effectiveness of the alpha particle, especially in PBFT. The utility of PBFT was verified using the simulation and it has a potential for application in radiotherapy.

## INTRODUCTION

Recently, the boron neutron capture therapy (BNCT) technique has gained acceptance as a method of treatment in the field of radiation therapy [1–3]. An alpha particle with high linear energy transfer (LET) is generated through a reaction between a neutron and a boron particle and can cause massive damage to a tumor cell. Tumor cells can be infused with boron through the injection of a boronate compound for PET scanning to assess the distribution of such as fluorine-borocaptate sodium (^18^F-BSH) or fluorine-paraboronophenylalanine (^18^F-BPA) and to estimate the boron concentration in the tumor before the neutron irradiation. Delivery of neutrons to this site will generate alpha particles through boron neutron capture solely in the tumor cells, and the alpha particles' short range corresponds to superior damage to the tumor while healthy tissue is spared [4, 5]. In other words, a large dose is delivered to the tumor by generating alpha particles within the tumor itself and not in healthy tissue. However, because neutrons themselves will damage normal tissue en route to the tumor, we believe an alternative method known as proton boron fusion therapy (PBFT) can be used for delivering a similar tumor dose distribution while reducing normal tissue dose compared to BNCT [6].

The proton boron fusion reaction was introduced in 1960 by many nuclear research groups. Three alpha particles are emitted after the reaction between a proton (^1^H) and a boron particle (^11^B). These three alpha particles can provide the damage to the tumor cell, just as in the case of alpha particles in BNCT. Theoretically, in the case of PBFT, the therapy efficacy per incident particle is three times greater than that of BNCT [7–11]. In addition, because the proton beam has the advantage of a Bragg-peak characteristic, normal tissue damage can be reduced. Since the previous study on BNCT, many studies for tumor treatment using alpha particles have been performed [11, 12]. In order to take advantage of alpha particles for dose delivery, two key points should be considered. First, the boron uptake should be labeled accurately to the target cell. As mentioned previously, alpha particles are generated where the boronate compound is accumulated. If this happens in normal tissue near the tumor region, alpha particles will damage the normal tissue as well as the tumor cell [13]. However, this problem has been resolved to a certain extent by many research groups. Thus, the chemical characteristics of boronate compounds and their biological effects were not considered in our study. Second, the number of generated alpha particles is also a significant factor for effective therapy. By using PBFT, a more effective therapy can be realized compared to BNCT or conventional proton therapy due to the large number of alpha particles generated, such as epithermal neutron.

Overall, BNCT is based on the neutron capture reaction. This reaction generates a lithium ion and an alpha particle (^10^B (n, α) ^7^Li). The emitted alpha particle can provide the damage to the tumor cell with the high linear energy transfer (LET). The reaction cross section between the boron and the thermal neutron is about 3840 barns (for 0.0025 eV neutron). After the injection of boronate compound to the tumor, the treatment is progressed through the external irradiation of thermal neutron beam. On the other hand, proton boron fusion therapy (PBFT) is based on the fusion reaction between the proton and the boron particle. Consequently, three alpha particles are emitted at the reaction point. These alpha particles provide more powerful damage to the tumor cell than only one alpha particle caused by the boron neutron capture reaction. The reaction cross section of proton boron fusion reaction is about 0.9 barn when the boron is an ion status with 675 keV resonant energy. In the concept of PBFT, the use of the boronate compound is almost same with the proceeding of BNCT. In PBFT, however, only ^11^B is required to react with the proton with particle status. Basically, BNCT use the ^10^B with an epithermal neutron (0.5 eV < En < 10 keV), is highly effective in capturing neutrons (2,500 times better than ^11^B, and eight times better than ^235^U). The PBFT is another method to capture the boron particle with stable 11B using a proton particle for releasing three alpha particles. The majority of the difference is the number of the alpha particle by bombarding the reaction. BNCT release an alpha particle (2.31 MeV) only. However, PBFT releases three alpha particles (3.74 MeV and 2.74 MeV). In addition, the intrinsic proton dose pattern follows Bragg-peak curve. The unnecessary irradiation can be reduced at the normal tissue regions.

To demonstrate the effectiveness of PBFT, we confirmed the dosimetric effects due to the proton boron fusion reaction in comparison to other cases. Thus, the purpose of this research was to verify the feasibility of PBFT by comparison to a BNCT simulation. Because the simulation study was calculated before the experiments, our research cannot provide comprehensive quantitative physical comparison data [12]. Some physical factors were simplified, and simulation variables were used without artificial controls. However, our research is the starting point for the development of a novel radiation therapy technique.

## METHODS AND MATERIALS

The concept of PBFT was suggested in our previous simulation study. The scope of the previous study was the amplification of peak integrated dose in the percentage depth dose (PDD). The reason for this amplification of peak integrated dose was discussed as the generation of alpha particles. Because the alpha particles include the proton beam, if the number of the alpha particle is increased, the number of protons also naturally increased. The scope of this simulation study was the verification of the contribution of alpha particles to the effectiveness of PBFT. All results were deducted by a Monte Carlo n-particle extended (MCNPX) simulation code. The simulation design for verification was constructed as four classes.

### Amplification of peak in the PDD

In order to extract the results of the PDD of the proton beam, a virtual phantom and the boron uptake region (BUR) were simulated. The size of the water phantom was set at 10 × 10 × 10 cm^3^, and the BUR size was 6 × 6 × 1 cm^3^. The energy of proton beam was varied from 75 MeV to 85 MeV with per 1 MeV. Because this simulation study was theory establishment level, the energy of the proton beam for clinical usage was not used. The phantom size was optimized to simulate the applied proton beam, and the location of the BUR was adjusted to the location of the peak integrated dose point. The boron concentration set at 1.04 mg/g. Because the amplification of peak integrated dose was difficult to observe when the clinical usage of boron concentration was used, a greater concentration usage was simulated. This means that the amount of amplification can be changed according to the concentration usage. Basically, all of the results of the first simulation were the PDDs of the proton beam. The absorbed dose of a proton was extracted from the water phantom including the BUR using the F6 tally in the MCNPX code [14, 15]. The tally frame was set at 1 mm (slab of water for extracting the absorbed dose). The proton beam was set at the point source to cover the BUR. A physics function in the MCNPX code was also considered to react with the boron particle. The direction of the beam is toward the BUR in the water. The distance between the source and the center of the water phantom was 60 cm. The peak integrated dose point can be located at almost the center of the phantom using this setting. For comparison, the PDD of an 80 MeV proton beam from the water phantom without the BUR was also acquired. Actually, the condition that can significantly contribute to the results is the reaction cross-section. In order to construct equivalent simulation conditions, we did not change the setting for the reaction cross-section, as recommended by the MCNPX user manual.

### Variation of proton dose by the boron neutron capture reaction

**Figure 1:**
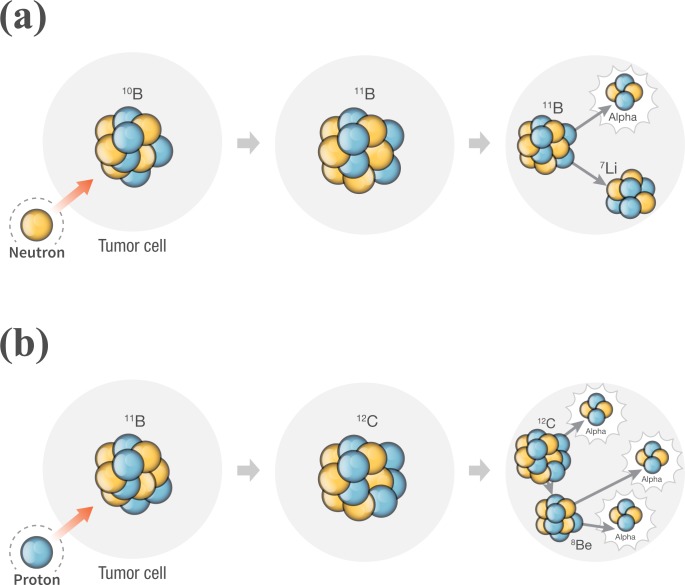
Diagram of generation principle of alpha particle through the nuclear reaction The top figure **a**. shows the alpha particle generation after the boron neutron capture reaction. The bottom figure **b**. is the generation of three alpha particles after the proton boron fusion reaction. The proton tally in the MCNPX can count the proton in the alpha particle.

The reason of the second simulation was to show the generation of the alpha particle indirectly. For alpha particles generated by the proton boron fusion reaction, the generation of alpha particles can be confirmed by the proton tally because the alpha particle includes the proton. Figure 1 shows the method for the prediction of the alpha particle generation by using the proton tally and the difference between the boron neutron capture reaction and the proton boron fusion reaction. The first simulation results were yielded based on the proton tally. The reason of amplification of peak integrated dose was difficult to describe using only the results of the first simulation. Under the same simulation conditions, we simulated the boron neutron capture reaction. The specifications of the phantom and BUR were fixed. The source was replaced with an epithermal neutron source (<0.0025 eV). When the center of BUR was located 3.5, 4.5, and 5.5 cm from the water surface, the variations in both the neutron and proton doses were verified. Each location of the BUR center has space to the thickness of the BUR. To display part of the main variation at the middle of the PDD, the above locations were used. In addition, the setting for reaction cross-section was not changed from the first simulation setting.

### Penumbra effect and prompt gamma ray energy spectrum

Gaussian, The PBFT method has some strong point from the dosimetric effect. We discovered a variation in the penumbra effect in the lateral dose profile when a proton reacted with a boron particle. To extract the lateral dose profile from the simulation, the lateral tally was set to the water phantom slab including the point of the amplified proton's maximum dose level. The lateral tally frame was also set at 1 mm. The simulation conditions were the same as the first simulation. The lateral profiles were extracted from the applied water slab (with/without the BUR) using the tally for an 80 MeV proton beam. In order to confirm the presence of low energy prompt gamma ray by the proton boron fusion reaction, the prompt gamma ray energy spectrum was extracted during the emission of the proton beam. For the spectrum, the F8 photon tally (energy deposition tally) in the MCNPX code was used. In addition, a detection system was added to the first simulation code. The detector material was chosen to be high purity germanium (HPGe, density= 5.32 g/cm^3^) for the energy resolution and detection efficiency regarding the prompt gamma ray peak on the energy spectrum [16]. The detector shape was a cylindrical shell type (Inner diameter= 30 cm, thickness= 5 cm). It surrounded the water phantom. The resolution setting was performed using the Gaussian energy broadening (GEB) function. The resolution references were 0.70% at 511 keV and 0.27% at 662 keV. We could compare the location of the prompt gamma ray peak to distinguish the boron neutron capture reaction and the proton boron fusion reaction.

## RESULTS AND DISCUSSIONS

Because the BNCT became well known in medical physics field, we especially concentrated on the phenomenon of the PBFT rather than the BNCT in this study. The proton beam energy chosen can cover approximately 4–5 cm in the water. In the actual clinical field, this range can be shortened by several factors. However, because the study step is a stage of establishment for the theory, to apply to a basic physical approach to the concept, data regarding the low energy proton beam are preferentially required for the simulation for clinical application step [17–19]. Thus, the phantom size and BUR specifications were adjusted to consider the proton beam energy level. When a higher energy is used, additional considerations should be generated.

**Figure 2:**
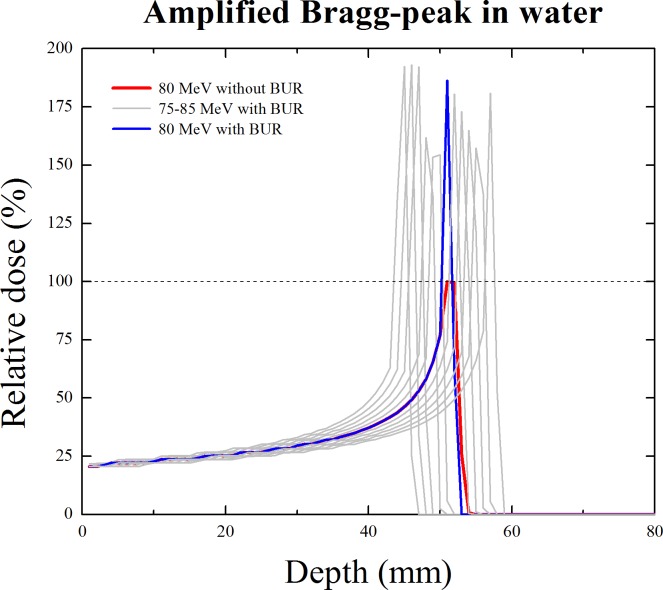
Amplification of peak integrated dose in the percentage depth dose (PDD) of proton The red line is the Bragg-peak curve of the proton from the water without a boron uptake region (BUR). The blue line shows the amplified Bragg-peak curve when the peak integrated dose point was located at the BUR. The grey line is also the amplified Bragg-peak curves of the various proton beam energies (from 75 to 85 MeV).

The PDDs including the amplified peak integrated dose are shown in Figure 2. The red line indicates the PDD of the proton beam from the water phantom without the BUR. The maximum dose level of that PDD set to 100%. It could be the standard for comparison with the amplified PDD by the proton boron fusion reaction. We observed that peak integrated dose is greater than the standard level (100%). When a boron concentration of 1.04 mg/g was used, the quantitative increase in the peak integrated dose is observed from the data listed in Table 1. As shown in Table 1, the peak value in the maximum dose level when the boron particle was accurately labeled at the region was 192.4% with 75 MeV.

**Table 1:** Maximum peak value of amplified Bragg-peak according to the proton beam energy

Energy (MeV)	Peak value (%)	Energy (MeV)	Peak value (%)
75	192.4	81	180.4
76	193.0	82	173.0
77	192.1	83	164.9
78	161.8	84	157.4
79	154.4	85	180.9
80	186.3		

The maximum value of normalized Bragg-peak through the 80 MeV proton beam from water without boron uptake region (BUR) is 100%.

The additional amplification of peak integrated dose was caused by the generation of alpha particles. In this case, more effective dose delivery to the target (tumor) is possible with unnecessary dose delivery to another part (normal tissue). Actually, this result is easily affected by the reaction cross-section and the boron concentration. Surely, we can observe a lower proton maximum dose level than standard according to these conditions. Because the original proton beam was used to react with boron, and the proton dose near the BUR depends on the generation of alpha particles, it does not mean that the therapy effect is lower than conventional proton therapy.

**Figure 3:**
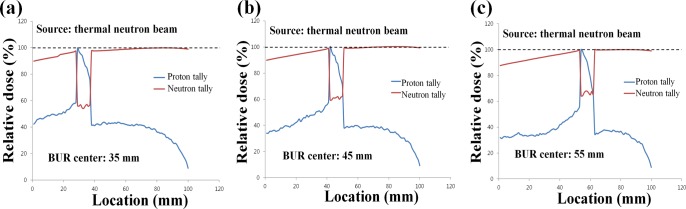
Percentage depth dose (PDD) of both neutron and proton after the boron neutron capture reaction The locations of the boron uptake region (BUR) are at 3.5 cm **a**., 4.5 cm **b**., and 5.5 cm **c**. from water surface. The red line shows neutron dose using neutron tally in the MCNPX. The blue line is the proton dose using proton tally during boron neutron capture reaction.

In order to add the proof of the influence by the alpha particle generation, the second simulation was performed. Figure 3 shows the variation in the neutron and proton doses from the water phantom including the BUR. The center of the BUR was located (a) 3.5 cm, (b) 4.5 cm, and (c) 5.5 cm from the water surface. The red line indicates the neutron dose, and the blue line indicates the proton dose. These two doses were extracted from one simulation simultaneously. In the case of the neutron dose, we can observe a dramatic decrease in the dose at the BUR because of the capture reaction. However, an increase in the proton dose at the BUR was also noted. After the reaction between boron and neutron, the alpha particles were generated, and these alpha particles were counted in the proton tally in the MCNPX. In the case of the proton tally, the shape of dose curve does seem like the ‘knife’. The number of generated alpha particles increases as a deeper relationship in the BUR. Because the first simulation results included the original proton beam's dose as well as the proton dose generated by the alpha particles, the proton dose amplification could be demonstrated.

**Figure 4:**
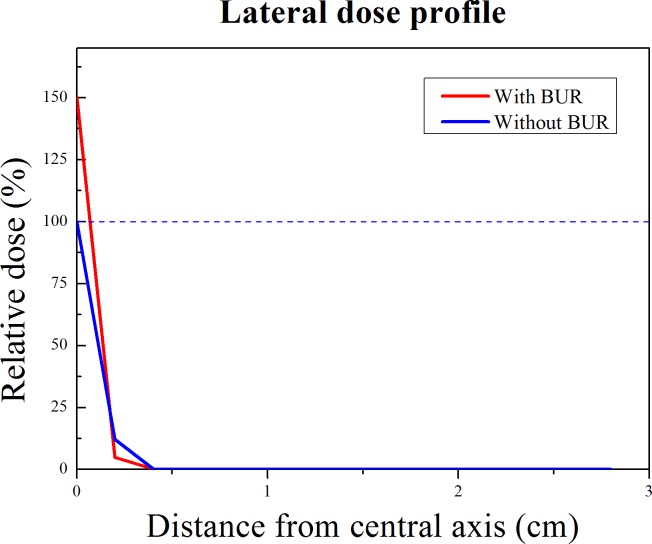
Lateral dose profile of 80 MeV proton beam from the water with/without boron uptake region The blue line is the dose profile of conventional proton beam from the water without the BUR. The red line shows the dose profile of proton beam at the amplified peak integrated dose point when the proton reacts with the boron particle. The effect of penumbra of red line is fewer than the blue line.

The purpose of third simulation was to show the one of strong point of PBFT. In the actual clinical field of proton therapy, the penumbra effect should also be considered. Effective therapy can be performed through a reduction in the penumbra effect. Figure 4 shows the lateral dose profiles of the proton from the water phantom with the BUR (red line) and without the BUR (blue line). Because of the proton boron fusion reaction, the maximum peak integrated dose of the red line is higher than that of the blue line. In addition, we could observe a clear difference in the penumbra region. The red line's penumbra region was sharper than the conventional PDD of the proton (blue line) beam. This result was caused by amplification of the proton's maximum dose level. This means that more accurate therapy than conventional proton therapy is possible. Surely, a synergistic effect can be expected with accurate boron uptake in the actual treatment, even though the difference is not strikingly large.

**Figure 5:**
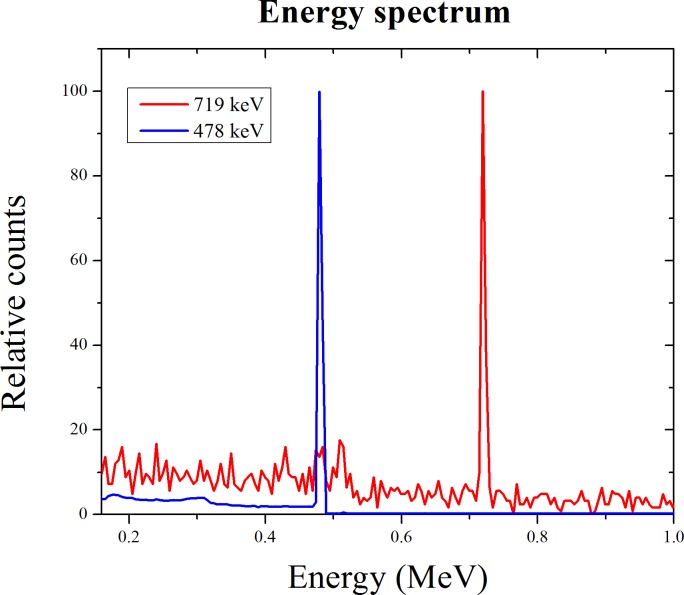
Energy spectra of prompt gamma ray generated by the nuclear reaction The blue line is the prompt gamma energy spectrum after the boron neutron capture reaction. The prompt gamma ray of 478 keV was appeared. The red line shows the prompt gamma ray peak of 719 keV from the proton boron fusion reaction.

In order to monitor the tumor status during BNCT, the prompt gamma rays generated by the capture reaction has been used. After the boron neutron capture reaction, a 478 keV prompt gamma ray is immediately emitted from the reaction point. It is possible to monitor the tumor status using prompt gamma rays during treatment [11]. Similarly, PBFT has the feasibility of tumor monitoring using prompt gamma ray imaging during treatment. When 478 keV gamma ray events are detected by the single photon emission computed tomography (SPECT) or the gamma camera, the tumor region can be monitored as the nuclear medicine imaging [20]. Similarly, we verified the prompt gamma ray event generated by the proton boron fusion reaction. The prompt gamma ray energy spectra are shown in Figure 5. The red line including the 478 keV energy peak is the photon energy spectrum of the boron neutron capture reaction, and the blue line is the photon energy spectrum of the proton boron fusion reaction. In order to use the prompt gamma ray event for nuclear medicine imaging, a low energy gamma ray event is required. At a result, we can observe the 719 keV energy peak. In case of the 719 keV energy peak, because it is classified as a high energy event, additional designs for the collimator and detector are required. If that construction is considered, the presence of a prompt gamma ray after the proton boron fusion reaction means that tumor monitoring is sufficient. This concept suggests a new direction for real-time tumor monitoring method during the PBFT. In the future, actual experiments will be performed to support our simulation results.

## CONCLUSION

From our previous study, the concept of PBFT was established. To verify the effectiveness of PBFT, a comparison study with a BNCT simulation was performed. We confirmed the sufficient utility of PBFT through this study. The PBFT has the advantages of both BNCT and proton therapy, and it can be a more effective therapy technique.
